# Assessment of Thrombotic and Bleeding Tendency in Two Mouse Models of Chronic Kidney Disease: Adenine-Diet and 5/6th Nephrectomy

**DOI:** 10.1055/s-0040-1705138

**Published:** 2020-04-16

**Authors:** Camélia Makhloufi, Lydie Crescence, Roxane Darbousset, Nathalie McKay, Ziad A. Massy, Christophe Dubois, Laurence Panicot-Dubois, Stéphane Burtey, Stéphane Poitevin

**Affiliations:** 1 Aix Marseille Univ, INSERM 1263, INRAE, C2VN, Marseille, France; 2 Division of Rheumatology, Immunology and Allergy, Brigham and Women's Hospital, Harvard Medical School, Boston, Massachusetts, United States; 3 Centre for Research in Epidemiology and Population Health (CESP), University Paris-Saclay, Villejuif, France; 4 Department of Nephrology, Ambroise Paré University Hospital, Boulogne Billancourt/Paris, France; 5 Centre de Néphrologie et Transplantation Rénale, APHM, Marseille, France

**Keywords:** tissue factor, platelets, hemostasis

## Abstract

The coexistence of bleeding and thrombosis in patients with chronic kidney disease (CKD) is frequent and poorly understood. Mouse models are essential to understand complications of CKD and to develop new therapeutic approaches improving the health of patients. We evaluated the hemostasis in two models of renal insufficiency: adenine-diet and 5/6th nephrectomy (5/6Nx). Compared with 5/6Nx mice, mice fed with 0.25% adenine had more severe renal insufficiency and so higher levels of prothrombotic uremic toxins like indoxyl sulfate. More severe renal inflammation and fibrosis were observed in the adenine group, as demonstrated by histological and reverse transcription quantitative polymerase chain reaction experiments. Liver fibrinogen γ chain expression and level of plasma fibrinogen were increased only in adenine mice. In both CKD mouse models, tissue factor (TF) expression was increased in kidney and aorta extracts. Immunochemistry analysis of kidney sections showed that TF is localized in the vascular walls. Thrombin–antithrombin complexes were significantly increased in plasma from both adenine and 5/6Nx mice. Tail bleeding time increased significantly only in adenine mice, whereas platelet count was not significant altered. Finally, results obtained by intravital microscopy after laser-induced endothelial injury showed impaired platelet function in adenine mice and an increase in fibrin generation in 5/6Nx mice. To summarize, adenine diet causes a more severe renal insufficiency compared with 5/6Nx. The TF upregulation and the hypercoagulable state were observed in both CKD models. Bleeding tendency was observed only in the adenine model of CKD that recapitulates the whole spectrum of hemostasis abnormalities observed in advanced human CKD.

## Introduction

It is well known that the incidence of cardiovascular events increases in patients with chronic kidney disease (CKD). 1 CKD patients exhibit many abnormalities in their hemostatic response that may account for their increased risk of both bleeding and thrombotic events. 2 The early stages of CKD are dominated by disturbances resulting likely in increased risk of myocardial infarction, peripheral arterial disease, stroke, and venous thromboembolism. In patients with advanced CKD, the procoagulant state persists, and in addition, uremia is strongly associated with platelet dysfunction leading to an increased risk of hemorrhagic events. 3 The pathogenesis of bleeding in uremia is considered multifactorial. The major defects involve primary hemostasis as anomalies in platelets–platelets and platelets–vessel wall interactions are of crucial importance. 4 Several clinical trials showed CKD as a strong risk factor for thrombosis independent of comorbidities such as diabetes, hypertension, and hypercholesterolemia. 5 Recent studies now strengthen an independent role of CKD in thrombosis by identifying uremia-specific prothrombotic risk factors. 6 7 8 9 Indeed, we and other have previously demonstrated the prothrombotic role of indolic uremic solutes indoxyl sulfate (IS) and indole-3 acetic acid. These indolic compounds induce tissue factor (TF) in both the endothelial cells and vascular smooth muscle cells (vSMCs). 6 8 10 11 In CKD patients, the balance of pro- and antithrombotic factors in blood is altered. Indeed, several prothrombotic hemostatic mediators are elevated, including fibrinogen, soluble thrombomodulin, soluble TF, thrombin–antithrombin TAT complex, von Willebrand factor, Factor VIII, and C-reactive protein (CRP). 2 12 The generalized inflammatory state, the endothelial dysfunction, and possibly poor clearance of some of the thrombotic mediators may account for this derangement. 5

The exact etiology behind the coexistence of thrombotic and bleeding risks in CKD is poorly understood. Animal models are therefore important tools to understand pathophysiological events occurring during kidney diseases. A better characterization of uremic mouse models will allow investigating the impact of specific target genes involved in hemostatic disorders related to CKD because of the availability of a large number of genetically engineered mouse strains.

## Materials and Methods

### Mice

C57BL/6 wild-type mice were purchased from Jackson Laboratories and maintained as a breeding colony in the animal care facility at the Faculty of Medicine of Marseille. Male mice were used in this study and comparisons were made between age-matched groups.

As a first model of CKD, the mice (13 weeks of age) were fed ad libitum with a 0.25% (w/w) adenine-enriched diet (A04 + 0.25% adenine, SAFE Augy, France) or a regular chow diet (A04 standard, SAFE) as controls ( Supplementary Fig. S1 ). After it was administered for 2 weeks to induce uremia, 13 adenine was withdrawn and the mice were fed with the regular chow diet for another 1 week. As a second model of CKD, we performed 5/6th nephrectomy (5/6Nx) using a two-step procedure with minor modifications, as previously described. 14 Briefly, mice (9-week-old) underwent 5/6Nx or sham surgery under general anesthesia (ketamine 125 mg/kg, xylazine 12.5 mg/kg, atropine 0.25 mg/kg; intraperitoneally [ *i.p.* ]). The left kidney was exposed through a flank incision, and we applied cortical electrocautery to the upper and lower poles (two-third of the left kidney). One week later, we performed right total nephrectomy through a similar incision. To prevent pain, buprenorphine (0.1 mg/kg) was injected (subcutaneously) before each surgery and in postoperative (day 1). All the surgical experiments were done by the same operator (SP). Mice were sacrificed at 6 weeks after induction of CKD. The experiments were performed in compliance with the Directive 2010/63/ EU of the European Parliament and were approved by the local Ethic Committee (Comité d'Ethique en Expérimentation Animale de Marseille, C2EA-14).

### Serum and Plasma Biochemistry

Depending on the dosages to be performed, mice blood was obtained during sacrifice either by puncture of the heart or by puncture of the vena cava under general anesthesia. Blood drawn via cardiac puncture was allowed to clot and was centrifuged to prepared serum. Platelet-free plasmas (PFP) were prepared from blood collected on 50 µL of sodium heparin (Héparine Choey, 250 UI/mL) via the inferior vena cava. Briefly, blood was centrifuged at 200 g/15min/20°C to obtain platelets-rich plasma (PRP). PRP was centrifuged at 2500 g for 15 minutes; the supernatant was collected and then centrifuged at 10,000 g for 10 minutes to obtained PFP. Aliquots of serum or PFP were stored at -80°C.

Creatinine and urea levels were measured with an Olympus AU400 autoanalyzer at the Biochemistry Laboratory of the Centre de Recherche sur l'Inflammation (UMR 1149 Inserm, Université Paris Diderot, ERL CNRS 8252, Paris, France). IS was measured by high-performance liquid chromatography using a reversed phase column, an ion-pairing mobile phase, and an isocratic flow, as described. 15

### RNA Extraction and RT-qPCR Analysis of mRNA Expression

Mouse liver, kidney (cortex and medulla), aorta, and heart were lysed in TRIzol and total ribonucleic acid (RNA) was purified by chloroform extraction and isopropanol precipitation. Reverse transcription (RT) was performed from 500 ng of total RNA with the Takara PrimeScript RT Reagent Kit (Ozyme, Saint Quentin en Yvelines, France). Primers and MGB-Taqman probes were purchased from ThermoFisher Scientific (Courtaboeuf, France) ( Supplementary Table S1 ). The polymerase chain reaction (PCR) reaction mixture was prepared using the Brilliant II QPCR Master Mix (Agilent Technologies, Les Ulis, France). All PCR reactions were performed in a Mx3000P (Agilent Technologies). The data were acquired and analyzed with the MxPro software (Agilent Technologies). Target gene expression was normalized on the basis of the hypoxanthine phosphorobosyltransferase (HPRT) content of each sample and was subsequently normalized to a basal mRNA level with the equation: N target = 2 ΔCt sample, where ΔCt is the Ct value of the target gene minus the Ct value of the HPRT gene. Normalized mRNA levels were determined by dividing the N target value by the N target value of the smallest quantifiable amount of target gene mRNA (target gene Ct value = 35). Data were then expressed as “fold induction” versus control.

### Immunohistochemistry

Paraffin sections of mouse kidneys (3.5 µm) were stained with Mayer's hematoxylin and eosin (H&E) (Sakura Finetek, France) or Picrosirius red (Sigma-Aldrich, Saint Quentin Fallavier, France) following kit instructions.

For TF immunohistochemistry, a goat antimouse TF (1/200, R&D Systems, Bio-Techne, Lille, France) was used. Antigen retrieval was performed using a sodium citrate buffer pH 6 (ThermoFisher) for 10 minutes at 120°C. Endogenous peroxidase activity was blocked with 3% H _2_ O _2_ and sections were blocked with 2% normal rabbit serum (Vector Laboratories). Primary antibody anti-TF was incubated overnight at 4°C. In parallel, a normal goat isotype control (R&D Systems, ref: AB-108-C) was used as a negative control. Sections were incubated with a rabbit biotinylated antigoat immunoglobulin (H ^+^ L) (1/1000, Vector Laboratories). After treatment with HRP Avidin D, then with the ImmPACT DAB Peroxidase (HRP) substrate (Vector Laboratories), sections were counterstained with Mayer's hematoxylin. Sample visualization was obtained using a LEICA DMRB microscope.

### Platelet Count

Platelets were count in PRP by flow cytometry ( Supplementary Fig. S2 ). The DyLight-488 conjugated platelet-specific (anti-glycoprotein Ib alpha [anti-GPIbα]) antibody (Emfret analytics, ref: X-488) was used to labeled platelets. Cell count was obtained by adding in samples a known number of Flow-Count Fluorospheres beads (Beckman-Coulter, France). The samples were acquired on a Gallios flow cytometer.

### Tail Bleeding Time

Mice were anesthetized (ketamine 125 mg/kg, xylazine 12.5 mg/kg, atropine 0.25 mg/kg; i.p.) and placed on a hot plate maintained at 37°C. Bleeding time assays were performed by cutting off the tip of the tail with a razor blade (5 mm from the tip). The tail was immediately immersed in saline solution heating at 37°C. The time to stable cessation of bleeding (no rebleeding within 30s) was visually determined. Bleeding was stopped manually at 5 minutes, if necessary, and mice that bled beyond this end point were counted as 5 minutes.

### ELISA

Commercial enzyme-linked immunosorbent assay [ELISA] kits from Abcam (Paris, France) were used to measure plasma (PFP) concentrations of fibrinogen (ref: ab108844) or TAT complexes (ref: ab137994). TF in aorta and kidney was measure by the commercial ELISA kit according to manufacturer's instructions (Abcam, ref: ab214091).

### Intravital Videomicroscopy

Mice were preanesthetized with i.p. injection of xylazine (12.5 mg/kg), ketamine (50 mg/kg), atropine (0.25 mg/kg) and maintained under anesthesia with repeated administration of pentobarbital following jugular vein catheterization. Tracheotomy was performed for facilitating respiration. Cremaster surgery preparation was carried through as previously described 16 and the muscle was superfused with thermocontrolled bicarbonate-buffered saline. Following antibodies were infused via jugular catheter as a single dose determined by the body weight of mice 17 : DyLight-488 or DyLight-649 conjugated platelet-specific (anti-GPIbα) (Emfret analytics, ref:X-488 and X-649) and DyLight-649 or DyLight-488 fibrin-specific (antifibrin) (provided by Pr. Bruce Furie, Harvard Medical School), respectively, for the adenine model and the 5/6Nx model. Real-time intravital microscopy in living mice was performed as previously described 16 using an Olympus AX microscope with a 60 × 0.9-numerical aperture water immersion objective. Fluorescence of platelet and fibrin were respectively and arbitrarily shown in green and red colors.

### Statistical Analysis

Data were expressed as mean ± SEM (standard error of the mean). Data from intravital videomicroscopy experiments were expressed as box plots of the indicated number of thrombi. The central box represents the values from lower to upper quartile (25th to 75th percentile) and the middle line represents the median. A line extends from the minimum to maximum value. Significant differences were revealed by the Mann–Whitney U-test performed with the Prism (GraphPad Software Inc, California, United States) software. *p* -Values less than 0.05 were considered as statistically significant.

## Results

### Adenine Diet Is a More Severe Model of CKD Than 5/6th Nephrectomy

As shown in the Table 1 , we first determined the change of body weight of mice compared with initial body weight at the beginning of protocols. Only adenine-fed mice exhibited a significant body weight reduction. All the other mice showed a weight gain compared with the beginning of the protocol and no significant difference was observed between the sham mice and the 5/6Nx mice. Blood urea nitrogen and serum creatinine are increased in both CKD mice models in comparison to their respective controls. Renal insufficiency is more severe in adenine than 5/6Nx mice group. The concentration of indoxyl sulfate, a well-studied uremic toxin, is increased in the both models of CKD. IS concentration in adenine-diet mice is similar to the concentration observed in patients with stage 5 CKD. In 5/6Nx mice, its concentration is closed to CKD stage 4. 7 These findings are consistent with renal impairment in both CKD models and demonstrated that CKD is more severe with the adenine diet in comparison to the surgical model of 5/6Nx.

**Table 1:** Body weight and biochemical findings in mice

	Normal diet	Adenine diet	Sham-operated	5/6Nx-operated
Change in body weight (%) a	105.6 ± 1.1	84.9 ± 1.1 ^b^	110.0 ± 0.7	105.1 ± 2.5
BUN (mmol/L)	7.9 ± 0.2	32.8 ± 2.5 ^b^	9.2 ± 0.3 ^c^	24.0 ± 2.6 ^d,e^
Creatinine (µmol/L)	27.5 ± 1.3	81.0 ± 4.4 ^b^	32.4 ± 1.1 ^c^	55.6 ± 2.1 ^d,f^
IS (µmol/L)	14.5 ± 2.0	161.8 ± 33.1 ^b^	12.9 ± 2.1	75.4 ± 29.8 ^d,e^

Abbreviations: BUN, blood urea nitrogen; IS, indoxyl sulfate; SEM, standard error of the mean.

a Compared with initial body weight at the beginning of protocols.

Note: Data are expressed as mean ± SEM ( *n* = 8/group). ^**b**^ *p* < 0.001 versus normal-diet group; ^c^ *p* < 0.05; ^**d**^ *p* < 0.001 *versus* sham-operated group; ^**d**^ *p* < 0.01 versus sham-operated group; ^e^ *p* < 0.05, ^f^ *p* < 0.001 versus adenine-diet group.

**Fig. 1:**
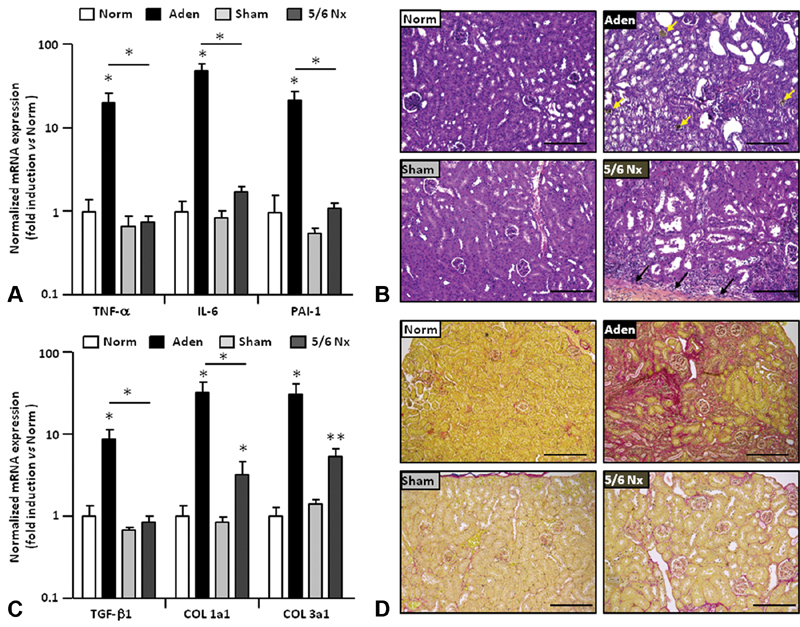
Renal inflammation and fibrosis in two models of mouse CKD. ( **A** ) Expression of *TNF-α, IL-6, and PAI-1* mRNA in the kidneys isolated from mice fed with adenine 0.25% (Aden) or with normal diet (Norm) and from sham- or 5/6Nx-operated mice. ( **B** ) Representative H&E staining of kidney sections. The yellow arrows point crystals of 2,8-dihydroxyadenine precipitated in tubules and the black arrows indicate the limit of injury causes by cauterization in residual kidney. ( **C** ) Kidney expression of *TGF-β1, COL1a1, and COL3a1* mRNA in mice fed with adenine 0.25% (Aden) or with Norm and from sham- or 5/6Nx-operated mice. ( **D** ) Representative Sirius red staining of kidney sections showing collagen deposition related to fibrosis. CKD, chronic kidney disease; *COL1a1* ; collagen type 1, alpha 1; 5/6Nx, 5/6th nephrectomy; H&E, hematoxylin and eosin; IL-6, interleukin-6; mRNA, messenger ribonucleic acid; PAI-1, plasminogen activator inhibitor-1; SEM, standard error of the mean; *TGF-β* 1; transforming growth factor-beta 1; TNF-α, tumor necrosis factor-alpha. Data are expressed as mean ± SEM, *n* = 4–6/group. * *p* < 0.05; ** *p* < 0.01. Scale bars = 200 μm.

We then investigated renal inflammation and fibrosis by reverse transcription quantitative polymerase chain reaction and histological analysis. A significant increase in the expression of tumor necrosis factor alpha *,* interleukin-6, and plasminogen activator inhibitor-1 ( *PAI-1* ) was observed in kidneys of mice from the adenine group ( Fig. 1A ) compared with the normal group. The expression of proinflammatory genes was unchanged in 5/6Nx mice versus sham-operated mice. In kidney of mice fed with adenine, H&E staining revealed a severe tubulointerstitial injury secondary to the precipitation of 2,8-dihydroxyadenine crystals ( Fig. 1B ) causing the renal inflammation. Pro-fibrotic genes *COL 1a1* and *COL 1a2* (respectively alpha 1 and alpha 2 chains of collagen type 1) were significantly upregulated in the two CKD mice models but the induction was lowered in 5/6Nx mice in comparison to adenine-fed mice ( Fig. 1C ). Renal transforming growth factor-beta 1 ( *TGF-β1* ) mRNA was significantly increased only in adenine-diet mice. Sirius red staining confirmed that renal fibrosis is more important in the adenine group that is supported by a dramatic increase in collagen deposition ( Fig. 1D ). The expression of proinflammatory and profibrotic genes was not induced in the liver of mice fed with adenine, confirming the specificity of the renal impairment in this CKD model ( Supplementary Fig. S3 ).

### Coagulation Is Activated in Both CKD Models

**Fig. 2:**
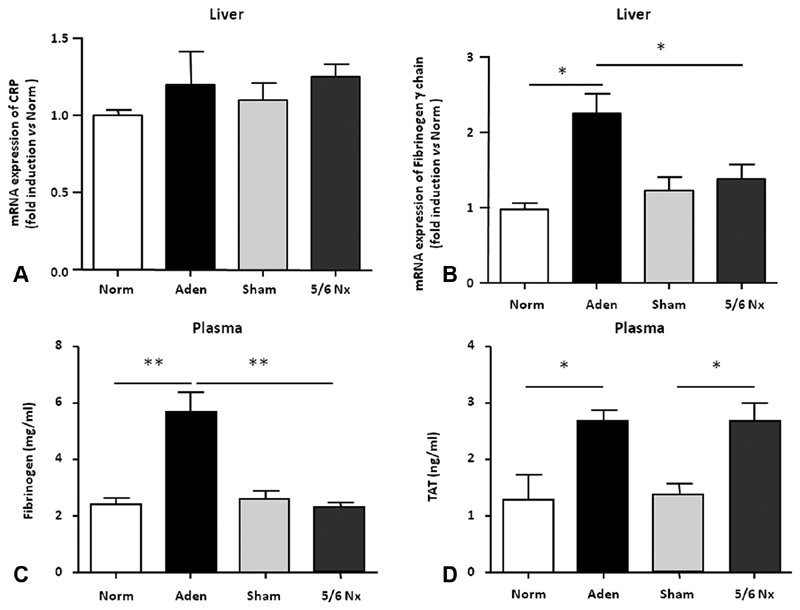
Systemic inflammation and hypercoagulability in mice with CKD. CRP ( **A** ) and fibrinogen γ chain mRNA expression ( **B** ) in the liver isolated from mice fed with adenine 0.25% (Aden) or with Norm and from sham- or 5/6Nx-operated mice. Fibrinogen ( **C** ) and TAT complexes ( **D** ) concentrations were measured in the plasma of CKD mice (Aden and 5/6Nx) and of their respective control (Norm and Sham). CKD, chronic kidney disease, CRP, C-reactive protein; mRNA, messenger ribonucleic acid; Norm, normal diet; Aden, adenine diet; Nx, nephrectomy; SEM, standard error of the mean; TAT, thrombi-antithrombin. Data are expressed as mean ± SEM, *n* = 4–6/group ( **A, B** ); *n* = 5–9/group ( **C, D** ). * *p* < 0.05; ** *p* < 0.01.

As coagulation and inflammation are closely linked, we first determined the expression of CRP and fibrinogen in the liver that could indicate systemic inflammation. CRP expression was low and not significantly upregulated in livers of CKD mice compared with their respective controls ( Fig. 2A ). In adenine-fed mice, there was a nearly twofold induction of fibrinogen γ chain expression in liver and of plasma fibrinogen ( Fig. 2B and 2C ) suggesting an elevation of systemic inflammation. Fibrinogen levels were similar in both sham and in 5/6Nx mice.

**Fig. 3:**
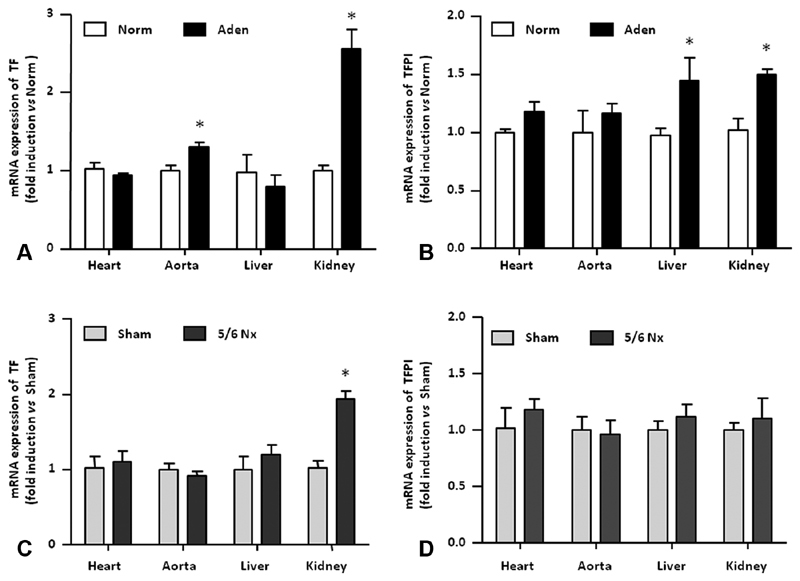
TF and TFPI mRNA expression in mice with CKD. TF expression ( **A, C** ) and TFPI expression ( **B, D** ) were measured in the Heart, the aorta, the liver, and kidneys isolated from CKD mice (Aden and 5/6Nx) and control mice (Norm and Sham). Aden, adenine diet; CKD, chronic kidney disease; mRNA, messenger ribonucleic acid; Norm, normal diet; Nx, nephrectomy; SEM, standard error of the mean TF, tissue factor; TFPI, tissue factor pathway inhibitor. Data are expressed as mean ± SEM, *n* = 5/group. * *p* < 0.05.

**Fig. 4:**
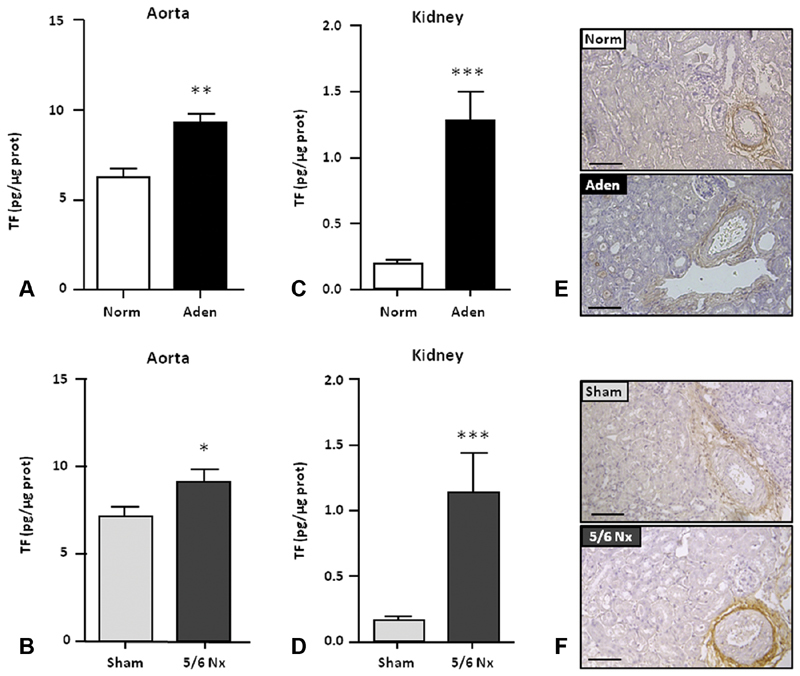
TF protein expression is increased in mice with CKD. ELISA of TF was performed with extract of aorta ( **A, C** ) and kidneys ( **B, D** ) isolated from mice fed with adenine 0.25% (Aden) or with Norm and from sham- or 5/6Nx- operated mice. ( **E, F** ) Representative immunohistochemical staining of TF in kidney. Aden, adenine diet; CKD, chronic kidney disease; ELISA, enzyme-linked immunosorbent assay; Norm, normal diet; Nx, nephrectomy; SEM, standard error of the mean; TF, tissue factor. Data are expressed as mean ± SEM, *n* = 6–8/group. * *p* < 0.05; ** *p* < 0.01; *** *p* < 0.001. Scale bars = 100 μm.

Abnormalities in coagulation in CKD patients are critical and represent the triggering of thrombotic events. We decided to investigate whether coagulation was activated in our two models of CKD mice by measuring plasma levels of TAT complexes. TAT levels were significantly increased (twofold) in adenine and 5/6Nx groups compared with their respective controls' mice ( Fig. 2D ). These results demonstrate that systemic hypercoagulability is similar in both mouse models of CKD. We next examined in distinct mouse organ the expression of both TF and tissue factor pathway inhibitor (TFPI), its physiologic inhibitor. *TF* mRNA expression was significantly increased in the aorta and in the kidney of adenine mice compared with control mice ( Fig. 3A ). *TFPI* mRNA levels were weakly and significantly induced in the liver and in the kidney of adenine mice ( Fig. 3B ). *TF* mRNA was increased only in the kidney of 5/6Nx mice and no difference in TFPI expression levels was observed in organs studied from sham and 5/6Nx mice ( Fig. 3C and D ). As determined by ELISA, TF protein was significantly increased in the aorta of adenine mice (by 1.5-fold) and of 5/6Nx mice (by 1.3-fold) compared with their respective controls ( Fig. 4A and B ). TF protein was also significantly increased in the kidneys of CKD mice from adenine and 5/6Nx groups (6.6- and 7.2-fold, respectively) compared with control groups ( Fig. 4C and D ). Immunohistochemistry analysis of kidneys obtained from CKD mice delineated a highly expression of TF protein in vascular wall ( Fig. 4E and F ). The TF immunolabeling was demonstrated as specific by the parallel use of a normal goat isotype control ( Supplementary Fig. S4 ). In kidney sections of adenine-fed mice, we also observed a TF protein expression in arterioles and peritubular capillaries ( Supplementary Fig. S5 ).

### Bleeding Phenotype Is Only Observed in Adenine-Diet-Induced CKD Mice

CKD patients are at high risk to experience hemorrhages. We explored thereby the bleeding tendency by measuring mouse tail bleeding time. Results showed that the bleeding time in the adenine group was significantly longer that in 5/6Nx mice and respective control mice ( Table 2 ). Nevertheless, the platelet counts were quite similar for both CKD mice models and control mice, excluding that the prolonged bleeding time observed in adenine mice group might be due to abnormal platelet number. Taken together, these results underlie a possible defect of platelet activation and/or function.

**Table 2:** Platelet counts and bleeding times in mice

	Normal diet	Adenine diet	Sham-operated	5/6Nx-operated
Bleeding time (s)	108.1 ± 18.8 ( *n* = 11)	254.3 ± 30.7 a ( *n* = 12)	91.6 ± 6.9 ( *n* = 18)	118.7 ± 13.4 b ( *n* = 19)
Platelets (x10 ^3^ /µL)	41.9 ± 9.9 ( *n* = 6)	31.9 ± 5.9 ( *n* = 6)	45.7 ± 9.1 ( *n* = 6)	37.9 ± 3.9 ( *n* = 6)

Abbreviation: SEM, standard error of the mean.

Note: Data are expressed as mean ± SEM.

a *p* < 0.01 versus normal-diet group.

b *p* < 0.01 versus adenine-diet group.

**Fig. 5:**
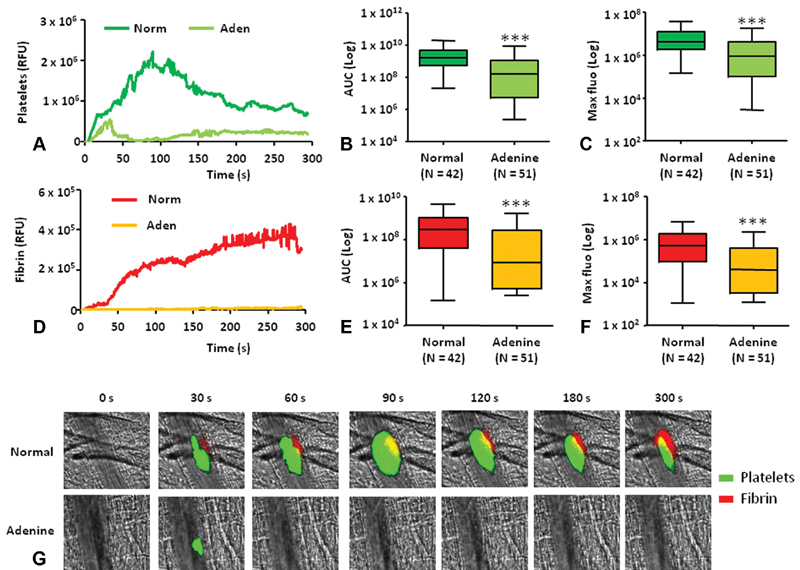
Thrombus formation following laser-induced injury is affected in the CKD model of mice fed with adenine. Kinetics of thrombus formation was assessed for 300 seconds after the initial injury of cremaster arterioles and median-integrated fluorescent intensities of normal-diet mice (dark green/red) and adenine-diet mice (light green/orange) after infusion of ( **A** ) anti-GPIbα (platelets) and ( **D** ) antifibrin (fibrin) are plotted over time. Data using either the maximum fluorescence ( **B** and **E** ) or AUC ( **C** and **F** ) are shown and are expressed as box plots of the indicated number of thrombi (N) obtained in three normal mice and four adenine mice. ( **G** ) Representative images of intravital microscopy are shown for each condition. *** *p* < 0.001. Aden, adenine; anti-GPIbα; anti-glycoprotein Ib alpha; AUC, area under the curve; CKD, chronic kidney disease; Norm, normal; RFU, relative fluorescence units. Scale bar =10 μm.

To confirm our investigations, we performed intravital microscopy experiments to track the formation of platelet thrombus in real time, in living animals. The analysis of kinetics of platelet accumulation revealed that platelet of adenine mice group had less ability to adhere, accumulate, and aggregate at the sites of the vascular injury in comparison to control group ( Fig. 5A and G ). Surprisingly, fibrin formation was also affected and mice under adenine diet were not able to stabilize the platelet clot with fibrin ( Fig. 5D and G ). Evaluation of the total platelet accumulation and fibrin generation by measuring the area under the curve (AUC) resulted in significant reduction in comparison to control ( Fig. 5B and E ). Similarly, the maximal platelet accumulation ( Fig. 5C ) and fibrin formation ( Fig. 5F ) were significantly decreased. These data are consistent with the bleeding time test findings previously described and confirm platelet function abnormalities and defect in fibrin formation in adenine mice group.

**Fig. 6:**
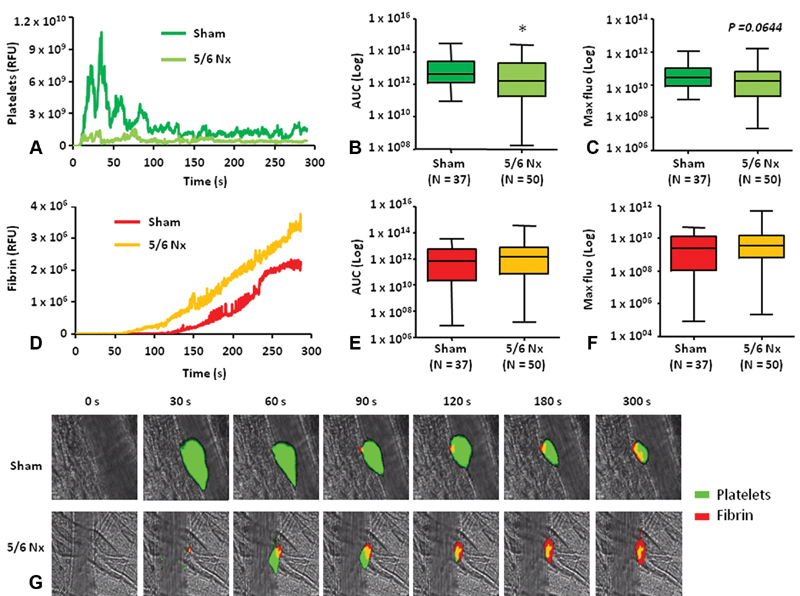
Thrombus formation following laser-induced injury is affected in the CKD model of 5/6th nephrectomy in mouse. Kinetics of thrombus formation was assessed for 300 seconds after the initial injury of cremaster arterioles and median-integrated fluorescent intensities of sham-operated mice (dark green/red) and 5/6- nephrectomy mice (light green/orange) after infusion of ( **A** ) anti-GPIbα (platelets) and ( **D** ) antifibrin (fibrin) are plotted over time. Data using either the maximum fluorescence ( **B** and **E** ) or AUC ( **C** and **F** ) are shown and are expressed as box plots of the indicated number of thrombi (N) obtained in five mice from each group. ( **G** ) Representative images of intravital microscopy are shown for each condition. * *p* < 0.05. anti-GPIbα; anti-glycoprotein Ib alpha; AUC, area under the curve; CKD, chronic kidney disease; Nx, nephrectomy; RFU, relative fluorescence units. Scale bar =10 μm.

We therefore assessed the kinetic of thrombus formation in CKD mice from the 5/6Nx model ( Fig. 6A-G ). The platelet accumulation and fibrin generation patterns were completely different as compared with normal mice reflecting the serious impact of surgical practice on animals ( Fig. 6A and D ). The measurement of AUC revealed a significant decrease in platelet accumulation in 5/6Nx mice compared with the sham-operated animals ( Fig. 6B ). No differences were observed when comparing the maximal platelet accumulation or fibrin generation ( Fig. 6C and F ). Nevertheless, a slight increase in fibrin formation was observed in 5/6Nx mice but was nonsignificant. These results can be explained by the presence of endogenous fibrin surrounding the vessel wall without any injury visible on screen during the acquisitions, which corroborate with the analysis of the fibrin generation kinetic that was found to be up to 1.5-fold higher in 5/6Nx mice group.

## Discussion

CKD is responsible for a thrombotic predisposition and a bleeding diathesis 18 that are associated with significant cardiovascular morbidity, severe hemorrhages, and mortality. Animal models are therefore necessary to mimic and to understand disturbances of hemostasis observed in human CKD to identify new therapeutic approaches. In the two animal models studied in this work, CKD mice displayed a hypercoagulable/prothrombotic state. Despite defect in platelets aggregation observed in the both models, the bleeding phenotype was observed only in CKD mice fed with adenine, after vessel injuries. These characteristics can be related to the severity levels of the renal insufficiency in the two CKD models studied. Indeed, mice from the adenine group had higher concentration of urea and creatinine levels compared with the mice with the 5/6Nx. All mice from this group exhibited body weight loss that we can explain by the low palatability of adenine diet for mice and consequently a reduced food intake as described in numerous recent studies. 19 20 21 A high adenine diet (ranging 0.2% to 0.3%) is known to induce CKD in mice via the development of tubulointerstitial nephropathy, 22 23 24 whereas 5/6Nx is regarded as a model of nephron reduction followed by glomerulosclerosis due to hyperfiltration injury. 25 In our study, kidney histology and PCR analysis are in accordance with the literature with an increase in renal inflammation and fibrosis in adenine-fed mice and increased glomerular collagen deposition in the remnant kidney of 5/6Nx mice.

CKD leads to the accumulation of uremic toxins having deleterious effect on multiple organs/systems. Among them, the indolic uremic toxins indoxyl sulfate (IS) is associated with overall and cardiovascular mortality in CKD patients. 26 In human, the mean IS uremic concentration is 109 µM and the highest uremic concentration measured is 210 µM. 27 IS concentrations increase during CKD progression to reach a mean concentration of 190 µM in end-stage renal disease (ESRD) patients 28 and IS level correlates with renal dysfunction markers (estimated glomerular filtration rate, creatinine, and urea). 29 Based on our biochemical analysis and on literature, we conclude that adenine model mimics human ESRD, whereas 5/6Nx model mimics less advanced stages of human CKD.

The nature of the renal disease induced by the two methods could also account for the differences observed in these models as 5/6Nx mimics nephronic reduction, while adenine diet rather induces an interstitial nephritis. In a model of acute kidney injury, mice fed with 0.25% adenine-enriched food for 10 days 30 and it was shown that renal failure was reversible in rat fed for 2 weeks with adenine then fed with normal diet for an additional 4 weeks. On the contrary, rats fed on the adenine diet for 4 weeks showed persistent renal dysfunction compatible to chronic renal failure seen clinically. 31 In a preliminary experiment, we observed 50% of mortality in mice fed with 0.25% adenine for 4 weeks (data not shown); therefore, mice were fed with 0.25% adenine for 2 weeks then with normal diet for 1 week. The mortality rate was then decreased at 22% but was higher than the mortality rate observed in the Nx model (15%; data not shown). Long-term reversibility after adenine withdrawal was not examined, but we hypothesized that 1 week of adenine withdraw correspond to a severe renal failure secondary to AKI. Consequently, renal damages, levels of renal failure, and mortality rates are closely related to the concentration of adenine in food and/or the duration of the diet and/or the duration of adenine withdrawal.

Concentration of plasma fibrinogen, a hepatic protein of the acute phase response, is reportedly increased and correlates in CKD patients with systemic markers of inflammation, notably the CRP. In addition, fibrinogen-γ chain mRNA is upregulated in the liver during inflammation. 32 Whereas CRP is a major acute-phase protein in human and rabbit, mouse CRP is not a major acute phase protein as determined by the measure of liver CRP mRNA concentrations after inflammatory stimuli. 33 Our data indicate that systemic inflammation is present in CKD mice following adenine diet but not in 5/6Nx mice, as demonstrated by the determination of both hepatic fibrinogen-γ chain mRNA and plasma fibrinogen.

We confirm also that CRP is not a good marker of inflammation in mice. No systemic effects of the standard high adenine diet, other than those mediated by kidney injury, have been reported. 34 We then conclude that systemic inflammation is the consequence of the renal injury in the adenine model and could participate in systemic hypercoagulation as determined by the increased plasma levels of TAT. In 5/6Nx mice, systemic hypercoagulation is observed in the absence of systemic inflammation. TAT levels were similar in both CKD mice models showing that the severity of renal insufficiency does not influence this parameter. In human, increased levels of TAT are among the main hemostatic abnormalities in patients with CKD. 3

We and others have demonstrated that TF, the main initiator of the coagulation, is increased in the plasma of patients with CKD 8 35 and that it is overexpressed in endothelial cells and vascular smooth muscle cells in response to various tryptophan-derived uremic toxin (TDUT) in vitro. 6 8 10 36 Here we show the TF increases in the aorta and in the wall of renal vessels in both types of CKD mice. In addition, we also observed renal expression of TF in arterioles and peritubular capillaries in adenine-fed mice highlighting the role of local inflammation in TF induction for this model. TF induction in vascular cells is an important prothrombotic mechanism increasing the risk of vascular injury–associated thrombosis as observed in CKD patients 37 and in animal models. 38 39

The impact of TDUT on thrombosis was recently studied in different murine models. Karbowska et al demonstrated that acute and chronic exposures to IS increase the weight of thrombi after vascular injury by electrical stimulation of the right common carotid artery in rat and increases thrombus formation after laser-induced endothelial injury in mice. 40 41 Kynurenine, another uremic solute, and IS increase thrombus formation in the carotid artery of mice in a model of ferric chloride (FeCl _3_ )-induced thrombosis. 28 37 In the same carotid artery thrombosis model, Yang et al linked IS-induced thrombosis with platelet hyperactivity in mice. 42 In contrast to these studies, we have studied hemostasis disturbances taking place in a global context of uremia in two CKD mice models displaying two different levels of uremia. Indeed, as CKD advances, the procoagulant persists, but in addition, patients start to exhibit platelet dysfunction that typically manifests with an increased risk of cutaneous, mucosal, or serosal bleeding. 3 Bleeding risk was evaluated using bleeding time. The formation of platelets thrombus was evaluated by intravital videomicroscopy after a laser-induced injury of cremaster arterioles. Adenine-fed mice display a much longer tail bleeding time than control mice and thrombus formation was strongly affected. Indeed, platelets adhere, accumulate, and aggregate poorly at the sites of the vascular injury and fibrin generation was significantly reduced in this mouse model of ESRD. It is interesting to note that the bleeding tendency was observed during surgical procedure for intravital microscopy experiments. In the less severe model of CKD, a significant decrease in platelet accumulation and an increase in fibrin generation were observed in 5/6Nx that could explain that the bleeding time was not significantly lengthened in these mice. The laser-induced injury model has been shown to stimulate thrombus formation without causing exposure of subendothelial collagens. 43 In this model, TF-mediated pathway of thrombin generation, by circulating microparticles and neutrophils, is the major pathway leading to platelet activation. 44 45 Our data indicate that blood-borne TF and platelet functions are affected in renal insufficiency. Platelet dysfunction beginning in less advanced stage of CKD is compensated by the hypercoagulable state induced by increased TF expression. In ESRD, the platelet dysfunction overcomes the activation of bleeding leading to a major bleeding risk. In rat with 5/6Nx and in mice fed with adenine for 2 weeks to induce CKD, an increase in thrombogenicity was shown in the carotid artery after FeCl _3_ -induced thrombosis. 28 46 This thrombosis model was shown to cause substantial damage to the endothelium causing exposure of underlying collagens and of vSMC–derived TF that is critical for arterial thrombosis in mice. 39 43 47 We hypothesize that thrombotic risk observed in CKD moderate stages and in ESRD is linked to arteries expressing vSMC-derived TF in response to various uremic toxins. TF is mainly responsible for thrombosis after vessel injury even if platelets are defective.

We recognize some limitations of the study. Some components of fibrinolysis known to be altered during CKD were not evaluated such as impaired release of tissue plasminogen activator or identified like increased levels of PAI-1. It would be also interesting to study “the new players in hemostasis and thrombosis” 48 such as TF-positive microparticles, neutrophils, or neutrophil extracellular traps in CKD mouse models.

Our results add new elements to understand the hemostatic disturbances during CKD progression and are complementary to previous studies. The hemorrhagic risk is mainly dependent on platelet dysfunction and occurs in the smallest diameter vessels such as arterioles where TF is poorly or not expressed and may reach larger vessels as renal failure progresses. In addition, these thrombosis and hemorrhagic processes occur with different kinetics and depend on the type of vascular injury and probably on fibrin clot structure as post-translational modifications of fibrinogen were reported in patients with CKD. 49 50 51

We confirmed, in mice models of renal insufficiency, that coagulation is activated mainly by TF increased expression and primary hemostasis is impaired mainly by platelets dysfunction. Several factors contribute to platelet dysfunction in patients with advanced CKD, such as impaired function of platelet glycoproteins, altered release of adenosine diphosphate and serotonin and faulty arachidonic acid and prostaglandin metabolism. Uremic toxins may contribute to platelet dysfunction by stimulating NO release. 3 Renal anemia may also play a pathogenetic role in the increased risk of bleeding in patients with advanced CKD because correcting it results in improved platelet function in this patient population.

The nature of the renal lesions and the severity of renal dysfunction play an important role to set the main phenotype observed in the models, moderate is mainly associated with hypercoagulable state, severe is associated mainly with bleeding risk. This is concordant with the observations in patients with CKD. To elucidate mechanism of thrombotic and bleeding tendency in CKD, we propose to use the adenine-diet model described in this work to study the bleeding risk associated with CKD and the 5/6Nx model to study the procoagulant phenotype.
