# Supplementary material for: Assessment of Thrombotic and Bleeding Tendency in Two Mouse Models of Chronic Kidney Disease: Adenine-Diet and 5/6th Nephrectomy
Source: TH Open. 2020 Apr 16;4(2):e66–76. doi: 10.1055/s-0040-1705138 (PMC7162676; doi:10.1055/s-0040-1705138)
Supplement: Supplementary file 1 — Supplementary Material [file 10-1055-s-0040-1705138-s190051.pdf]

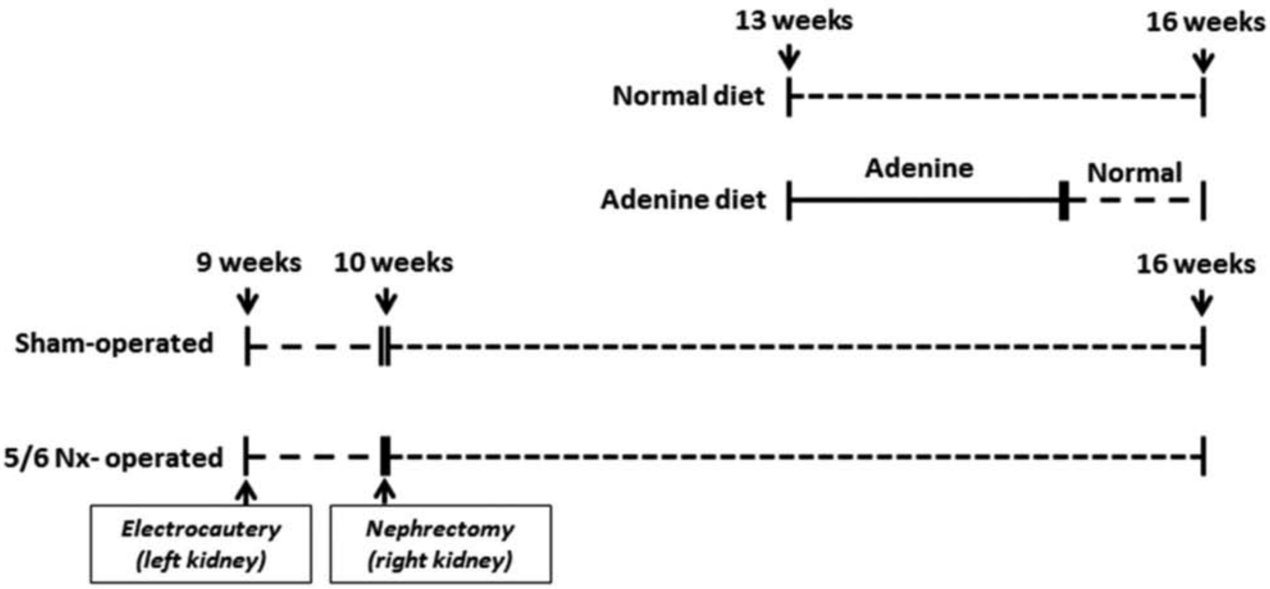

Supplemental Fig. S1 Timeline and design of two CKD mouse models (adenine diet and ⅚ nephrectomy).

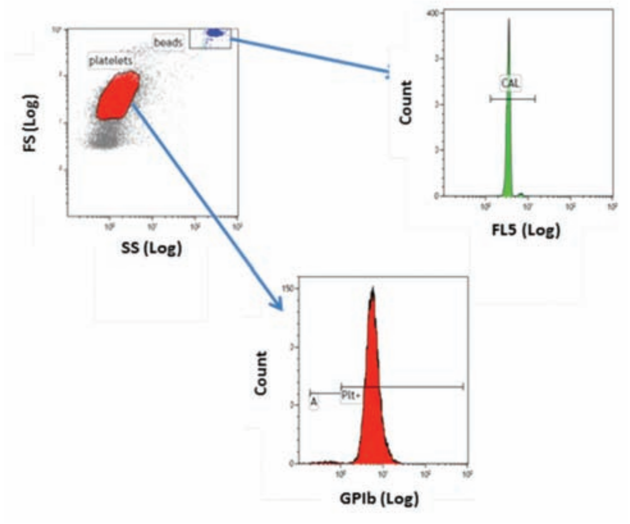

Supplemental Fig. S2 Platelets count in mouse plasma by flow cytometry. Platelets labeled with platelet-specific (anti-GPIb) antibody (Plt +) were count through a known number (CAL) of beads added to samples and acquired in parallel (FL5 fluorescence).

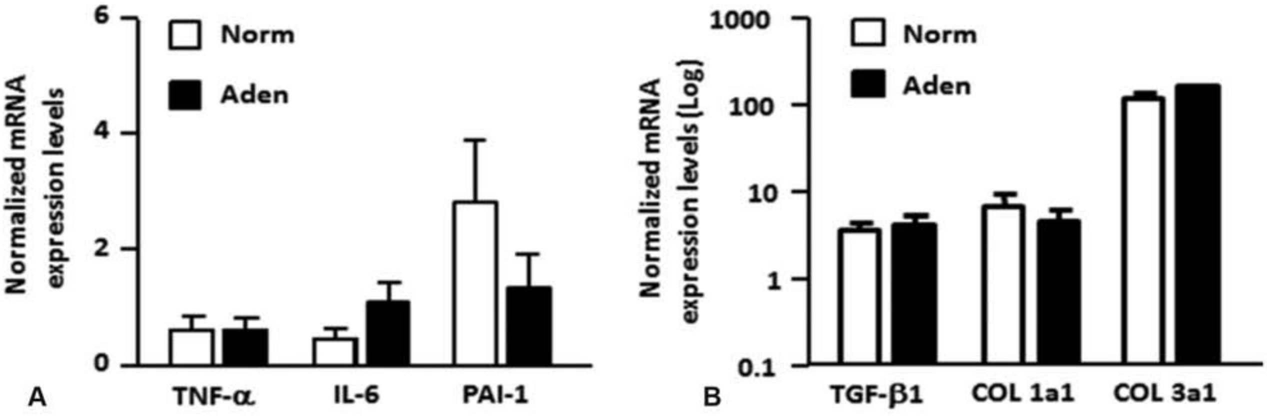

Supplemental Fig. S3 The adenine diet does not cause inflammation ((A)) or fibrosis ((B)) in the liver of mice. Expressions of proinflammatory genes (*TNF-a, IL-6 and PAI-1*) and profibrotic genes (*TGF-b1, COL1a1 and COL 3a1*) were determined in the liver isolated from mice fed with adenine 0.25% (Aden) or with normal diet (Norm). Data are expressed as mean ± SEM, $n = 4$/group.

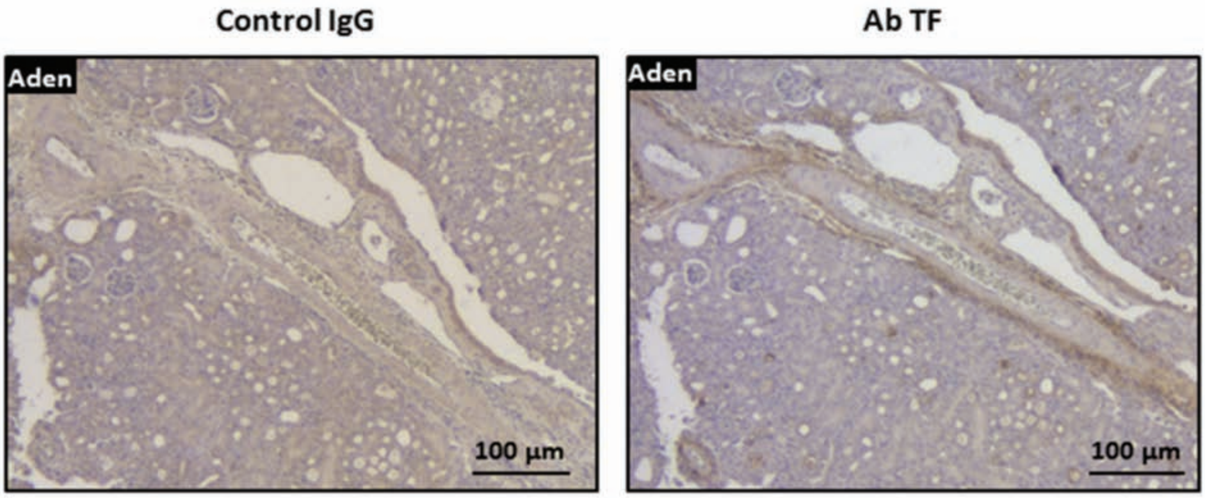

Supplemental Fig. S4 Specificity of Tissue factor (TF) labeling in kidney section from adenine fed mouse (Aden) in comparison with control IgG.

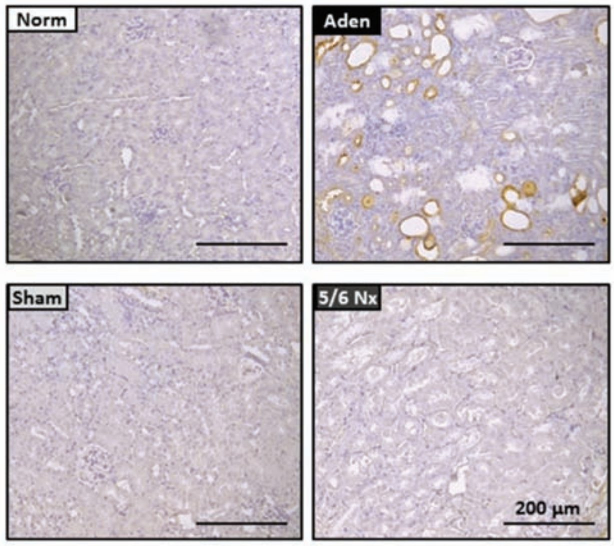

Supplemental Fig. S5 Representative immunohistochemical staining of TF in kidney isolated from mice fed with adenine 0.25% (Aden) or with normal diet (Norm) and from sham- or ⅚ Nx- operated mice. Abbreviation: Nx, nephrectomy.

Supplemental Table S1 List of primer references used in qPCR experiments

Target genes	TaqMan probes ID
HPRT	Mm01545399_m1
TNF-α	Mm00443258_m1
IL-6	Mm00446190_m1
PAI-1	Mm00435860_m1
TGF-β1	Mm01178820_m1
COL 1a1	Mm00801666_g1
COL 3a1	Mm01254476_m1
Fibrinogen γ chain	Mm00513575_m1
Tissue Factor (TF)	Mm00438853_m1
TFPI	Mm01334601_m1
